# Incidence of Osteoarthritis Between ACL Reconstruction With Different Graft Types and Between ACL Reconstruction and Repair: A Systematic Review and Meta-analysis of Randomized Controlled Trials

**DOI:** 10.1177/23259671241258775

**Published:** 2024-08-14

**Authors:** Tom Vendrig, Michèle N.J. Keizer, Reinoud W. Brouwer, Roy A.G. Hoogeslag

**Affiliations:** † Center for Human Movement Sciences, University Medical Center Groningen, University of Groningen, Groningen, the Netherlands; ‡ Department of Orthopedic Surgery, Martini Hospital, Groningen, Groningen, the Netherlands; § Centre for Orthopaedic Surgery and Sports Medicine OCON, Hengelo, the Netherlands; Investigation performed at the Center for Human Movement Sciences, University Medical Center Groningen, University of Groningen, Groningen, the Netherlands

**Keywords:** allograft, anterior cruciate ligament reconstruction, anterior cruciate ligament repair, bone-patellar tendon-bone autograft, hamstrings tendon autograft, osteoarthritis, quadriceps tendon autograft

## Abstract

**Background:**

Variation in stiffness, fixation methods, and donor-site morbidity after anterior cruciate ligament reconstruction (ACLR) with different graft types and with anterior cruciate ligament suture repair (ACLSR) can lead to differences in dynamic knee laxity and consequent differences in posttraumatic osteoarthritis (PTOA) development.

**Purpose:**

To compare the incidence of PTOA between different graft types used for primary ACLR and between primary ACLR and ACLSR. It was hypothesized that the incidence of PTOA would vary between ACLR with different autografts and allografts and between ACLR and ACLSR.

**Study Design:**

Systematic review; Level of evidence, 1.

**Methods:**

A search of the literature was performed to identify all randomized controlled trials (RCTs) comparing radiographic evidence of PTOA after ACLR between different graft types—hamstring tendon (HT) autograft, bone-patellar tendon-bone (BPTB) autograft, quadriceps tendon autograft, and allograft—and between ACLR and ACLSR. The minimum follow-up was 2 years. Study quality was assessed using the modified Coleman Methodology Score. A meta-analysis was performed to determine whether there was a difference in the incidence of PTOA between the different graft types and between ACLR and ACLSR.

**Results:**

Eleven randomized controlled trials were included in the meta-analysis—HT: 440 patients (mean follow-up, 9.7 years); BPTB: 307 patients (mean follow-up, 11.8 years); allograft: 246 patients (mean follow-up, 5 years); ACLSR, 22 patients (5 years). No study reporting the incidence after ACLR with quadriceps tendon was included. The study quality ranged from 70 to 88. The meta-analysis indicated no significant difference in the incidence of PTOA between graft types used for ACLR and between ACLR and ACLSR (risk ratios: HT vs BPTB, 1.05; HT vs allograft, 0.81; BPTB vs allograft, 0.82; HT vs ACLSR, not estimable [*P* > .05 for all]). The combined number of patients with PTOA in all studies per graft type showed that patients who underwent ACLR with a BPTB autograft had the highest percentage of PTOA (HT, 23.4%; BPTB, 29.6%; allograft, 8.1%; ACLSR, 0%). However, excluding studies with a follow-up <5 years resulted in similar outcomes for patients with an HT autograft and a BPTB autograft.

**Conclusion:**

This meta-analysis reported no difference in the incidence of PTOA between graft types used for ACLR and between ACLR and ACLSR. More research is necessary to make a reliable conclusion about which technique is associated with the lowest incidence of PTOA after ACL surgery.

Anterior cruciate ligament (ACL) injuries are becoming more common because of an increase in sports participation, and they often lead to knee instability, reduced activity levels, and impaired quality of life.^16,17,26,54^ ACL injuries can be treated with ACL surgery to regain knee stability; nonetheless, knee instability may still occur after surgery.^18,40^ This might be one of the reasons that the incidence of posttraumatic osteoarthritis (PTOA), which is 50% to 90% in the long term,^36^ is not reduced after ACL surgery compared with conservative treatment.^13^ PTOA causes knee pain and stiffness, leading to limitations of daily activities, deterioration of mental health, and reduced quality of life.^24,29,43,44^

Widely used graft types for ACL reconstruction (ACLR) include hamstring tendon (HT), bone-patellar tendon-bone (BPTB), and quadriceps tendon (QT) autografts and allografts.^58^ These grafts all have different stiffness levels.^12,32,49,52^ Furthermore, the choice of graft type also affects fixation strength. One of the reasons for this is that fixations of grafts with a bone block (BPTB on both sides, QT on one or no side) are stronger than fixations of grafts with a tendon-to-bone fixation (HT on both sides, QT on one or both sides).^3^ Moreover, the harvest site location of these autografts has an impact on the hamstring-quadriceps strength ratio; patients with an HT autograft have reduced hamstring strength, while patients with a BPTB autograft or a QT autograft have reduced quadriceps strength.^38,51^ During dynamic movements, larger quadriceps and smaller hamstring forces can reduce knee stability in ACL-deficient knees.^50^ Patients with an allograft do not experience potential changes in dynamic knee stability caused by donor-site morbidity. Furthermore, in the last decade, interest in ACL suture repair (ACLSR) has reawakened because of improved insight into biologics and biomechanics of the ruptured ACL.^19,21,22,41^ Patients treated with ACLSR also do not experience donor-site morbidity, and an animal study even showed that the short-term development of PTOA after ACL repair was less than after ACLR.^27^

The variation in stiffness, fixation methods, and donor-site morbidity of different ACLR graft types and ACLSR might lead to a difference in residual and dynamic knee laxity after ACL surgery.^31,50^ Increased knee laxity can result in abnormal loading conditions of knee cartilage compared with healthy knees, which can contribute to the development of PTOA.^2,7^ As such, the incidence of PTOA may also vary after different surgical techniques. To the best of our knowledge, no systematic review has included all autograft types, different allografts, and ACLSR to examine the difference in incidence of PTOA after the surgical treatment of a ruptured ACL. Therefore, this study aimed to perform a systematic review to critically appraise, summarize, and compare randomized controlled trials (RCTs) on the incidence of PTOA between ACLR with different autografts and allografts and between ACLR and ACLSR. We hypothesized that the incidence of PTOA would vary between ACLR with different autografts and allografts and between ACLR and ACLSR.

## Methods

### Search Strategy and Inclusion Criteria

The PRISMA (Preferred Reported Items for Systematic Reviews and Meta-Analysis) guidelines were followed to conduct this systematic review. This study was registered in the International Prospective Register of Systematic Reviews (PROSPERO) (CRD42023395623). A single reviewer (T.V.) performed a literature search in the PubMed, Cochrane, Embase, and CINAHL databases up to October 2022. Key search terms included “ACL,”“anterior cruciate ligament,”“osteoarthritis,”“cartilage,”“radiographic,”“hamstrings,”“semitendinosus,”“gracilis,”“patellar,”“bone-patellar tendon-bone,”“quadriceps,”“allograft,”“anterior cruciate ligament repair,” and “ACL repair.” The titles and abstracts were reviewed for all search results. Potentially eligible studies were reviewed in full text (T.V. and M.J.N.K.). In addition, the reference lists of the included articles were screened for potentially eligible studies.

The inclusion criteria were studies that (1) were clinical RCTs, (2) compared primary ACLR with at least 2 graft types (HT autograft, BPTB autograft, QT autograft, allograft) or (3) primary ACLR with any of the mentioned graft types and primary ACLSR, (4) performed a baseline assessment of cartilage damage, (5) reported postoperative radiological evidence of PTOA (radiograph or magnetic resonance imaging) based on any type of classification system, (6) had a minimum 2-year follow-up after surgery, and (7) were published in English after 2005. The inclusion of studies was regardless of the reported surgical technique regarding tunnel placement or graft fixation technique. The exclusion criteria were (1) studies that did not report the number or percentage of patients per grade of PTOA according to a classification system at the follow-up, (2) studies that included patients with severe multiligament injuries, and (3) animal studies. In the case of 2 studies reporting an identical patient group with different follow-up lengths, only the study with the longer follow-up was included. In the case of 2 studies containing a (partially) overlapping patient group, only the study with the largest patient group was included.

### Data Extraction

If reported, the following data were collected from each study: number of participants, age, surgical technique, duration of follow-up, inclusion and exclusion criteria, postoperative rehabilitation protocol, method of radiological evaluation for classification and grading of PTOA, and number or percentage of patients per grade of the used classification system for grading PTOA.

### Evaluation of Study Quality

The modified Coleman Methodology Score (MCMS) was used to examine the methodological quality of the included studies,^11^ of which ranged from 0 to 100. A score of <55 is considered poor, 55 to 69 is fair, 70 to 84 is good, and 85 to 100 is excellent.

### Meta-analysis

A meta-analysis was performed to determine whether there was a difference in the incidence of PTOA between patients after ACLR with different graft types and between patients after ACLR and ACLSR. PTOA was defined as scoring a grade ≥2 at the follow-up on the Kellgren-Lawrence (KL) classification system, scoring a grade ≥1 at the follow-up on the Ahlbäck classification system, or scoring a grade ≥C at the follow-up on the radiograph findings subsection of the objective International Knee Documentation Committee (IKDC) knee examination form, as described by Claes et al.^10^ Data analysis was performed with RevMan 5.4 statistic software (Cochrane Collaboration). The risk ratio with a 95% CI was used as the summary statistic to perform statistical analysis. Effect sizes were pooled using fixed-effects models, with significance set at *P* < .05. Statistical heterogeneity was tested with the chi-square and *I*^2^ tests, with significance set at *P* < .10.

In addition, the combined number of patients with PTOA from all studies was calculated separately for each graft type and ACLSR, and these differences were compared with the chi-square test. Also, a subgroup analysis was performed for studies with a minimum follow-up duration of 5 years. The independent variable was the group (HT autograft, BPTB autograft, allograft, ACLSR), and the dependent variable was the incidence of PTOA (number of patients with PTOA, number of patients without PTOA). The significance level was set at *P* < .05.

## Results

### Characteristics of the Included Studies

**Figure 1.**
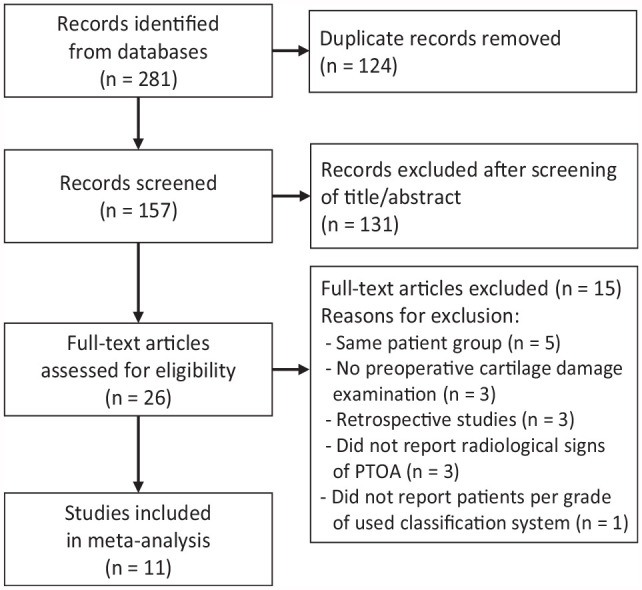
Flowchart of the literature search process. PTOA, posttraumatic osteoarthritis.

Eleven studies^‖^ were included in this systematic review (Figure 1 and Table 1). No studies have compared the incidence of PTOA after ACLR with a QT autograft or any other graft type or ACLSR. The mean follow-up period for all studies was 12.9 years (range, 7-17 years). All studies used radiographs exclusively to report postoperative evidence of PTOA. To grade PTOA, 3 studies^4,8,57^ used the KL classification, 2 studies^1,8^ used the Ahlbäck and Fairbank classification, and 2 studies^39,46^ used the radiograph findings subsection of the objective IKDC knee examination form.^59^

**Table 1:** Study Characteristics and Outcomes of Included Studies^*a*^

Lead Author (Year)	Technique Groups	No. of Patients^*b*^	Patient Age, Years	Follow-up, Years	Outcomes		*P*
					Technique 1, %	Technique 2, %	
HT autograft vs BPTB autograft							
Ahldén^1^ (2009)	3ST/4ST vs BPTB	23 vs 21	26	7.3	HT autograft • Ahlbäck score (medial): 8.7 grade 1, 4.3 grade 5 • Ahlbäck score (lateral): 4.3 grade 3 • Fairbank: 70 PTOA	BPTB autograft • Ahlbäck score (medial): 9.5 grade 1 • Ahlbäck score (lateral): 9.5 grade 1 • Fairbank: 67 PTOA	NS for all
Barenius^4^ (2014)	4ST vs BPTB	65 vs 69	40^*c*^	14.1	HT autograft • KL score (medial): 65 grade ≥2	BPTB autograft • KL score (medial): 49 grade ≥2	NS
Björnsson^8^ (2016)	3ST/4ST/STG vs BPTB	86 vs 61	27	16.5	HT autograft • KL score: 29.1 grade 1, 26.7 grade 2, 9.3 grade 3, 4.7 grade 4 • Ahlbäck score (medial): 17.4 grade 1, 7 grade 2, 1.2 grade 3, 1.2 grade 4 • Ahlbäck score (lateral): 19.8 grade 1, 2.3 grade 2, 1.2 grade 3 • Mean cumulative Fairbank score grade change = 2.1	BPTB autograft • KL score: 23 grade 1, 36.1 grade 2, 9.8 grade 3, 3.3 grade 4 • Ahlbäck score (medial): 24.6 grade 1, 4.9 grade 2, 1.6 grade 3 • Ahlbäck score (lateral): 24.6 grade 1, 4.9 grade 2, 1.6 grade 3 • Mean cumulative Fairbank score grade change = 2.4	NS for all
Matsumoto^39^ (2006)	STG-BP vs BPTB	35 vs 37	24	7	HT autograft • IKDC score: 28.6 grade B, 8.6 grade C	BPTB autograft • IKDC score: 32.4 grade B, 8.1 grade C	NS
Sajovic^46^ (2018)	STG vs BPTB	24 vs 24	25	17	HT autograft IKDC score: • 5 y: 18 grade B • 11 y: 52 grade B, 7 grade C, 4 grade D • 17 y: 50 grade B, 13 grade C, 8 grade D	BPTB autograft IKDC score: • 5 y: 46 grade B, 4 grade C • 11 y: 40 grade B, 44 grade C • 17 y: 67 grade B, 21 grade C, 12 grade D	<.05 for all
Webster^57^ (2016)	4ST vs BPTB	19 vs 19	26	15.3	HT autograft • KL score: 68 grade 0-1, 32 grade 2-3	BPTB autograft • KL score: 74 grade 0-1, 26 grade 2-3	NR
HT autograft vs allograft							
Tian^55^ (2016)	STG vs STG-IR	40 vs 43	29	6.9	HT autograft • KL score: 7.5 grade 1, 5 grade 2, 2.5 grade 3 • Versus preop: 7.5 ↑ 1 KL grade, 2.5 ↑ 2 KL grades	Allograft • KL score: 20.9 grade 1, 9.3 grade 2, 4.7 grade 3 • Versus preop: 20.9 ↑ 1 KL grade, 11.6 ↑ 2 KL grades	<.05
Tian^56^ (2016)	STG vs STG-AL	62 vs 59	30	4.6	HT autograft • KL score: 8.1 grade 1, 4.8 grade 2, 1.6 grade 3 • Versus preop: 6.5 ↑ 1 KL grade, 4.8 ↑ 2 KL grades	Allograft • KL score: 8.5 grade 1, 5.1 grade 2, 1.7 grade 3 • Versus preop: 8.4 ↑ 1 KL grade, 3.4 ↑ 2 KL grades	NS
Yoo^61^ (2017)	STG vs TA	68 vs 64	27	2.8	HT autograft • KL score: 19.1 grade 1, 2.9 grade 2 • Versus preop: 7.4 ↑ 1 KL grade	Allograft • KL score: 18.8 grade 1, 1.6 grade 2 • Versus preop: 6.3 ↑ 1 KL grade	NS
BPTB autograft vs allograft							
Sun^53^ (2009)	BPTB vs BPTB-AL	76 vs 80	32	5.6	BPTB autograft • KL score: 39 grade 1, 7 grade 2, 3 grade 3 • Versus preop: 38 ↑ 1 KL grade, 9 ↑ 2 KL grades	Allograft • KL score: 39 grade 1, 8 grade 2, 4% grade 3 • Versus preop: 36 ↑ 1 KL grade, 10 ↑ 2 KL grades	NS
HT autograft vs ACLSR							
Hoogeslag^23^ (2022)	4ST vs dynamic augmented ACLSR	18 vs 22	21	5	HT autograft • KL score: 22.2 grade 1	ACLSR KL score: 9.1 grade 1	NS

a The given age of patients is at the time of surgery unless otherwise indicated. Patient age and follow-up are reported as means. ACL, anterior cruciate ligament; ACLSR, anterior cruciate ligament suture repair; BPTB, bone-patellar tendon-bone autograft; BPTB-AL, nonirradiated fresh-frozen bone-patellar tendon-bone allograft; 4ST, quadrupled semitendinosus autograft; HT, hamstring tendon autograft; IKDC, International Knee Documentation Committee; KL, Kellgren-Lawrence; NR, not reported; NS, not significant; PTOA, posttraumatic osteoarthritis; preop, preoperative; STG, semitendinosus and gracilis autograft; STG-AL, nonirradiated fresh-frozen semitendinosus and gracilis allograft; STG-BP, semitendinosus and gracilis autograft with bone plugs on both sides; STG-IR, irradiated fresh-frozen semitendinosus and gracilis allograft; TA, tibialis anterior allograft; 3ST, 3-strand semitendinosus autograft; ↑, improvement.

b Number of patients is shown for each surgical technique group, respectively.

c Mean age at the follow-up was reported.

Six studies^1,4,8,39,46,57^ compared ACLR with an HT autograft versus a BPTB autograft. A total of 483 patients were examined, of which 252 patients underwent ACLR with an HT autograft and 231 patients underwent ACLR with a BPTB autograft. Five studies reported the age of the patients at surgery (mean, 26 years; range, 14-59 years). One study^4^ reported the age of the patients at the follow-up (mean, 40 years). Two studies^4,57^ used a quadrupled semitendinosus tendon as the HT autograft; 1 study^1^ used either a 3-strand or quadrupled semitendinosus tendon as the HT autograft depending on the length and diameter of the tendon; 1 study^8^ used either a 3-strand semitendinosus, a quadrupled semitendinosus, or a 4-strand semitendinosus and gracilis (STG) tendon as the HT autograft and considered patients receiving these tendons as a single group; one study^39^ used a 5-strand STG tendon with bone plugs placed at both ends; and 1 study^46^ used an STG tendon as the HT autograft. The femoral tunnel was drilled using a transtibial tunnel technique in 3 studies,^1,39,57^ with an accessory anteromedial portal technique in 2 studies,^4,47^ and with both methods in 1 study.^8^ All studies excluded patients when moderate or severe cartilage damage was reported preoperatively, except 1 study^39^ in which cartilage damage was observed in 14% of the patients in both groups. Postoperative rehabilitation protocols were similar across studies, except for 2 studies^39,46^ that used bracing during the first weeks of rehabilitation and 2 studies^4,57^ that did not report the rehabilitation protocol.

Included were 3 studies^55,56,61^ that compared ACLR with an HT autograft versus an allograft. A total of 336 patients (mean age, 29 years; range, 13-62 years) were examined in these 3 studies, 170 underwent ACLR with an HT autograft, and 166 underwent ACLR with an allograft. These studies excluded patients with moderate or severe cartilage damage preoperatively (an Outerbridge grade >2 in 2 studies^55,56^ and a KL grade >1 in 1 study^61^) and used an STG autograft as the HT autograft. Tian et al^55^ used an irradiated fresh-frozen STG allograft in 1 RCT and a nonirradiated fresh-frozen STG allograft in another RCT,^56^ and Yoo et al^61^ used a nonirradiated fresh-frozen tibialis allograft. The femoral tunnel was drilled with an accessory anteromedial portal technique in all 3 studies.^55,56,61^ The mean follow-up was 4.8 years (range, 2.8-6.9 years). All 3 studies used the KL classification system^59^ to grade PTOA at the follow-up.

One study^53^ compared ACLR with a BPTB autograft versus an allograft. A total of 156 patients were examined, of whom 76 patients underwent ACLR with a BPTB autograft and 80 patients underwent ACLR with a fresh-frozen nonirradiated BPTB allograft. The femoral tunnel drilling technique was not reported. The mean age of the patients was 32 years (range, 19-65 years). Patients with moderate or severe preoperative cartilage damage were not excluded. Before surgery, 4% of patients who underwent ACLR with a BPTB autograft and 5% of patients who underwent ACLR with an allograft had Outerbridge grades of ≥3 osteoarthritis. The mean follow-up of this study was 5.6 years (range, 4-8 years). The KL classification was used to grade PTOA at the follow-up.

One study^23^ compared ACLR with an HT autograft versus ACLSR. A total of 40 patients were examined, of which 18 patients underwent ACLR with a quadrupled HT autograft with an all-inside technique, and 22 patients underwent ACLSR using the dynamic intraligamentary stabilization method.^15^ The femoral tunnel was drilled with a retrograde drill technique in patients who underwent ACLR, with an accessory anteromedial portal technique in patients who underwent ACLSR. The mean age of the patients was 21 years (range, 10-27 years). Patients with moderate or severe preoperative cartilage damage were excluded. The mean follow-up of this study was 5 years, and the KL classification was used to grade PTOA at the follow-up.

### Evaluation of Study Quality

Table 2 presents the level of evidence and methodological quality of the included studies according to the MCMS. The study quality ranged from 70 to 88, with 5 studies^8,53,55,56,61^ considered excellent and 6 studies^1,4,23,39,46,57^ considered good. Two studies^4,57^ failed to report the rehabilitation process after surgery, and 1 study^4^ did not provide a description of the surgical technique. Two studies^23,57^ had a relatively small patient group, and 1 study^46^ did not report whether the assessor was independent.

**Table 2:** Methodological Quality and Level of Evidence of the Included Studies^*a*^

Study (Year)	MCMS Score	Level of Evidence
HT autograft vs BPTB autograft	Mean, 78.3	
Ahldén et al^1^ (2009)	78	NR
Barenius et al^4^ (2014)	73	1
Björnsson et al^8^ (2016)	86	2
Matsumoto et al^39^ (2006)	84	1
Sajovic et al^46^ (2018)	79	2
Webster et al^57^ (2016)	70	1
HT autograft vs allograft	Mean, 86.7	
Tian et al^55^ (2016)	86	2
Tian et al^56^ (2016)	86	2
Yoo et al^61^ (2017)	88	1
BPTB autograft vs allograft		
Sun et al^53^ (2009)	88	2
HT autograft vs ACLSR		
Hoogeslag et al^23^ (2022)	82	1
Total	Mean, 81.8	

a ACLSR, anterior cruciate ligament suture repair; BPTB, bone-patellar tendon-bone; HT, hamstring tendon; MCMS, modified Coleman Methodology Score; NR, not reported.

### Outcomes

All but 2 studies^46,55^ reported no significant differences between the study groups. Sajovic et al^46^ reported a significant difference with a higher incidence of PTOA after ACLR with a BPTB autograft compared with an HT autograft at 5-, 12-, and 17-year follow-ups. Tian et al^55^ reported a significant difference with a higher incidence of PTOA after ACLR with an HT allograft compared with an HT autograft at a 7-year follow-up. No significant difference in the incidence of PTOA was reported after ACLR with a BPTB autograft versus an allograft or after ACLR with an HT autograft versus ACLSR. The outcomes of these studies are presented in Table 1.

### Results of Meta-analysis

**Figure 2.**
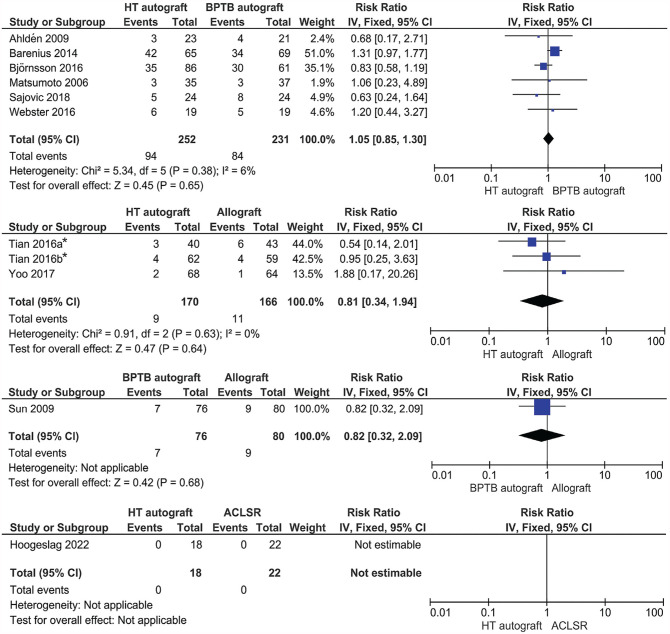
Comparison of the results of PTOA between graft types and between ACLR and ACLSR. *Tian (2016a) is reference 55, and Tian (2016b) is reference 56. ACLR, anterior cruciate ligament reconstruction; ACLSR, anterior cruciate ligament suture repair; BPTB, bone–patellar tendon–bone; HT, hamstring tendon; IV, inverse variant; PTOA, posttraumatic osteoarthritis.

The results of the meta-analysis are presented in Figure 2. No significant difference was found in the incidence of PTOA between graft types used for ACLR and between ACLR and ACLSR (risk ratios: HT vs BPTB, 1.05; HT vs allograft, 0.81; BPTB vs allograft, 0.82; HT vs ACLSR, not estimable [*P* > .05 for all]). No studies comparing ACLR with a QT autograft with another graft or ACLSR were included in this analysis.

### Combined Number of Patients With PTOA

The combined number of patients with PTOA from all studies for each graft type and for ACLSR are presented in Table 3, along with the number and mean follow-up period of the included studies. The ACLSR group was excluded from the statistical analysis because of a small patient group, and ACLR with a QT autograft was not included in this analysis because of a lack of studies.

**Table 3:** Combined Patients With PTOA From all Studies for Each ACLR Graft Type and for ACLSR^*a*^

Surgical Technique	No. of Studies	Follow-up, y, Mean (Range)	PTOA, % (n/Total)
Studies with minimum 2-year follow-up			
HT autograft	10	9.7 (2.8-17)	23.4 (103/440)
BPTB autograft	7	11.8 (5.6-17)	29.6 (91/307)
Allograft	4	5 (2.8-6.9)	8.1 (20/246)
ACLSR	1	5 (NR)	0 (0/22)
Studies with minimum 5-year follow-up			
HT autograft	8	11.1 (5-17)	31.3 (97/310)
BPTB autograft	7	11.8 (5.6-17)	29.6 (91/307)
Allograft	2	6.3 (5-6.9)	12.2 (15/123)
ACLSR	1	5 (NR)	0 (0/22)

a ACLR, anterior cruciate ligament reconstruction; ACLSR, anterior cruciate ligament suture repair; BPTB, bone-patellar tendon-bone; HT, hamstring tendon; NR, not reported; PTOA, posttraumatic osteoarthritis.

Including the patients of all studies with a minimum follow-up of 2 years, a significant difference was reported in the combined number of patients with PTOA of all studies between graft types for ACLR (χ^2^[2, n = 993] = 39; *P* < .05). The highest percentage of patients with PTOA occurred after ACLR with a BPTB autograft, and the smallest percentage of patients with PTOA occurred after ACLR with an allograft. The subgroup analysis—including only patients of studies with a minimum follow-up duration of 5 years—also reported a significant difference in PTOA between ACLR graft types (χ^2^[2, n = 740] = 17.4; *P* < .05).

## Discussion

The most important finding of this study was that the meta-analysis showed no significant difference in the incidence of PTOA between ACLR graft types and between ACLR and ACLSR. Because of the absence of literature about the incidence of PTOA after ACLR with a QT autograft, there was no comparison made between this graft type and other graft types or ACLSR.

In contrast to these findings, a meta-analysis by Xie et al^60^ and a systematic review by Poehling-Monaghan et al^45^ reported a higher incidence of PTOA after ACLR with a BPTB autograft compared with an HT autograft. The difference in the outcome of the present meta-analysis as well as the studies of Xie et al and Poehling-Monaghan et al might be explained by the inclusion of retrospective studies, whereas the present study performed a systematic review and analysis of only RCTs. Whereas Poehling-Monaghan et al did not perform a meta-analysis of the included studies, the present study included 4 recent studies that compared HT autografts versus BPTB autografts that were published after the search period of Xie et al. Therefore, the present meta-analysis includes more recent studies that have a higher level of evidence. A systematic review by Belk et al^6^ also reported no difference in the incidence of PTOA between patients who underwent ACLR with an HT autograft versus a BPTB autograft. The present study was an extension of the study of Belk et al, with additional studies that used the IKDC classification system to grade PTOA. Furthermore, the present study included other graft types used for ACLR and also compared ACLR with ACLSR.

No differences in the incidence of PTOA after ACLR with an allograft versus an autograft were reported in this meta-analysis. However, there might be a difference between different allografts. While Tian et al^55^ in 1 study reported a significantly higher incidence of PTOA after ACLR with an irradiated HT allograft compared with an HT autograft, Tian et al^56^ in another study and Yoo et al^61^ reported no difference between an allograft and an HT autograft when the allografts were not irradiated. The irradiation of the allograft may have decreased its stiffness, leading to decreased knee stability and a higher incidence of PTOA.^32^ In the study of Tian et al,^55^ patients who underwent ACLR with an irradiated HT allograft had decreased anterior and rotational knee stability compared with patients who underwent ACLR with an HT autograft. The decreased stiffness of the irradiated allograft could have had a negative impact on the knee stability, which may have led to a higher incidence of PTOA.

The literature comparing the incidence of PTOA after ACLSR versus ACLR is scarce, with only 1 study^23^ with a relatively small patient group being included in this meta-analysis, which did not report a significant difference. Recent studies have reported promising results regarding ACLSR,^19,22,41^ as it seemed to be noninferior to ACLR in terms of knee laxity. Karamchedu et al^27^ showed a lower incidence of PTOA in animals treated with ACL repair compared with ACLR. Furthermore, a recent RCT by Barnett et al^5^ reported better results on the Knee injury and Osteoarthritis Outcome Score (KOOS)-Symptoms subscale 1 year after surgery in patients who underwent ACLSR compared with patients who underwent ACLR. The study of Barnett et al, together with previous studies of Leister et al^34^ and Murray et al,^42^ have reported noninferior outcomes on the other KOOS subscales. Thus, promising outcomes related to the progression of PTOA after ACLSR have been reported, but very few studies of the actual incidence after ACLSR exist.

No studies were found comparing the incidence of PTOA after ACLR with a QT autograft versus other graft types or ACLSR with a minimum 2-year follow-up. Numerous studies have examined PTOA-related outcomes in patients who underwent ACLR with a QT autograft. Lee et al^33^ reported radiographic progression of PTOA in patients who underwent ACLR with a QT autograft, which increased from 19.4% before surgery to 33.8% at the final follow-up of 10 years (KL grade, ≥1). When comparing the increase in the incidence reported by Lee et al, with the combined number of patients with PTOA from all studies included in the present study (Table 3), a QT autograft seems to have an advantage over other grafts with the same follow-up period. However, Martin-Alguacil et al,^37^ who obtained ultrasound imaging measurements 1 year after surgery in patients who underwent ACLR with a QT autograft and an HT autograft, reported similar results between the 2 grafts. Moreover, several recent studies have compared the KOOS outcomes of patients who underwent ACLR with a QT autograft with other grafts^¶^; and of these 9 studies, 8 studies^14,20,25,28,30,35,48,62^ reported that having a QT autograft does not cause patients to have inferior KOOS outcomes. Cavaignac et al^9^ reported that patients who underwent ACLR with a QT autograft had better KOOS-Symptoms and KOOS-Sport scores compared with patients who underwent ACLR with an HT autograft; however, no difference was reported in the other KOOS subscales. Therefore, similar to ACLSR, ACLR with a QT autograft shows promising results related to the progression of PTOA, but comparisons of the incidence of PTOA with other graft types are still scarce.

The combined number of patients with PTOA compared every graft type with each other, including all patients of studies with a minimum follow-up of 2 years, and with a subgroup analysis including only patients of studies with a minimum follow-up of 5 years. This resulted in a larger pool of patients compared with the results of the meta-analysis that only examined 2 graft types with each other. Including all patients of studies with a minimum follow-up of 2 years, the combined number of patients with PTOA per graft type for ACLR showed the highest incidence of PTOA in patients after ACLR with a BPTB autograft and the lowest incidence of PTOA in patients after ACLR with an allograft. However, in the subgroup analysis of only studies with a minimum 5-year follow-up, the difference in the incidence of PTOA between a BPTB and an HT autograft became minimal, which might be the result of a more similar mean follow-up duration of the included studies. Nevertheless, we found significant differences between graft types, likely attributable to the small number of patients with PTOA after ACLR with an allograft. Although the mean follow-up duration of the included studies that examined patients with an allograft increased in the subgroup analysis, it was still notably lower compared with studies that examined patients with an HT autograft and a BPTB autograft. PTOA is a degenerative joint disease, and it takes time to develop and progress; thus, the short mean follow-up duration of patients with an allograft is highly likely to have influenced these results and should therefore be interpreted with caution.

### Limitations

Several limitations of this review should be addressed. No reliable conclusion about which graft type for ACLR or ACLSR resulted in the lowest incidence of PTOA could be made, because, respectively, little or no studies that investigated the incidence of PTOA after ACLSR and ACLR with a QT could be included. Furthermore, to create a larger pool of patients, all the included studies were combined to calculate the number of patients with PTOA per graft type and ACLSR. This resulted in a large variability in the follow-up duration, the number of studies and patients, and the mean age at surgery between graft types for ACLR and ACLSR. Because of this heterogeneity, the results of this analysis could not make a reliable comparison between all surgical options after ACL injury. In particular, the results of the incidence of PTOA after ACLSR should be interpreted carefully, as only 1 study^23^ with a small and young patient group and a midterm follow-up period was included. Furthermore, there was a large variation in surgical techniques regarding femoral tunnel placements, graft fixation, and graft preparation between included studies, which led to further heterogeneity. Last, a minimum follow-up duration of 2 years can be considered short term with regard to the assessment of PTOA. However, this was done to prevent neglecting the already scarce high-quality studies that contain valuable information regarding PTOA after ACLR with allografts and ACLSR. Therefore, a subgroup analysis was performed, including only studies with a minimum follow-up of 5 years. It would be interesting for future studies to report the incidence of PTOA, including all graft types for ACLR and ACLSR in 1 RCT with a long-term follow-up. In particular, the incidence of PTOA after ACLR with a QT autograft and after ACLSR is currently not or hardly investigated, while promising results regarding KOOS scores, donor-site morbidity, and knee laxity have been reported.

## Conclusion

The findings of this meta-analysis indicated no difference in the incidence between graft types used for ACLR and between ACLR and ACLSR. The number of patients with PTOA was similar after ACLR with an HT autograft and a BPTB autograft. The number of patients with PTOA after ACLR with an allograft was lower compared with other graft types; however, the available studies contributing to this analysis were limited in number, and their follow-up durations were relatively short. RCTs that compared the incidence of PTOA after ACLSR and ACLR with a QT autograft with other graft types used for ACLR and ACLSR were scarce and are necessary to make a reliable conclusion about which technique results in the lowest incidence of PTOA after ACL surgery.

## Notes

‖ References 1, 4, 8, 23, 39, 46, 53, 55-57, 61.

¶ References 9, 14, 20, 25, 28, 30, 35, 48, 62.

## References

[1] Ahldén M Kartus J Ejerhed L Karlsson J Sernert N. Knee laxity measurements after anterior cruciate ligament reconstruction, using either bone-patellar-tendon-bone or hamstring tendon autografts, with special emphasis on comparison over time. Knee Surg Sports Traumatol Arthrosc. 2009;17(9):1117-1124. 19575180 10.1007/s00167-009-0846-5

[2] Andriacchi TP Briant PL Bevill SL Koo S. Rotational changes at the knee after ACL injury cause cartilage thinning. Clin Orthop Relat Res. 2006;442:39-44. 16394737 10.1097/01.blo.0000197079.26600.09

[3] Aune AK Ekeland A Cawley PW. Interference screw fixation of hamstring vs patellar tendon grafts for anterior cruciate ligament reconstruction. Knee Surg Sports Traumatol Arthrosc. 1998;6(2):99-102. 9604194 10.1007/s001670050080

[4] Barenius B Ponzer S Shalabi A , et al. Increased risk of osteoarthritis after anterior cruciate ligament reconstruction: a 14-year follow-up study of a randomized controlled trial. Am J Sports Med. 2014;42(5):1049-1057. 24644301 10.1177/0363546514526139

[5] Barnett SC Murray MM Badger GJ , et al. Earlier resolution of symptoms and return of function after bridge-enhanced anterior cruciate ligament repair as compared with anterior cruciate ligament reconstruction. Orthop J Sports Med. 2021;9(11):23259671211052530. 10.1177/23259671211052530 PMC8581796 34778483

[6] Belk JW Kraeutler MJ Carver TJ McCarty EC. Knee osteoarthritis after anterior cruciate ligament reconstruction with bone-patellar tendon-bone versus hamstring tendon autograft: a systematic review of randomized controlled trials. Arthroscopy. 2018;34(4):1358-1365. 29366740 10.1016/j.arthro.2017.11.032

[7] Beveridge JE Proffen BL Karamchedu NP , et al. Cartilage damage is related to ACL stiffness in a porcine model of ACL repair. J Orthop Res. 2019;37(10):2249-2257. 31125133 10.1002/jor.24381 PMC6739195

[8] Björnsson H Samuelsson K Sundemo D , et al. A randomized controlled trial with mean 16-year follow-up comparing hamstring and patellar tendon autografts in anterior cruciate ligament reconstruction. Am J Sports Med. 2016;44(9):2304-2313. 27229354 10.1177/0363546516646378

[9] Cavaignac E Coulin B Tscholl P , et al. Is quadriceps tendon autograft a better choice than hamstring autograft for anterior cruciate ligament reconstruction? A comparative study with a mean follow-up of 3.6 years. Am J Sports Med. 2017;45(6):1326-1332. 28273424 10.1177/0363546516688665

[10] Claes S Hermie L Verdonk R Bellemans J Verdonk P. Is osteoarthritis an inevitable consequence of anterior cruciate ligament reconstruction? A meta-analysis. Knee Surg Sports Traumatol Arthrosc. 2013;21(9):1967-1976. 23100047 10.1007/s00167-012-2251-8

[11] Coleman BD Khan KM Maffulli N Cook JL Wark JD. Studies of surgical outcome after patellar tendinopathy: clinical significance of methodological deficiencies and guidelines for future studies. Victorian Institute of Sport Tendon Study Group. Scand J Med Sci Sports. 2000;10(1):2-11. 10693606 10.1034/j.1600-0838.2000.010001002.x

[12] Cornu O Banse X Docquier PL Luyckx S Delloye C. Effect of freeze-drying and gamma irradiation on the mechanical properties of human cancellous bone. J Orthop Res. 2000;18(3):426-431. 10937629 10.1002/jor.1100180314

[13] Cuzzolin M Previtali D Zaffagnini S , et al. Anterior cruciate ligament reconstruction versus nonoperative treatment: better function and less secondary meniscectomies but No difference in knee osteoarthritis—a meta-analysis. Cartilage. 2021;13(suppl 1):S1658-S1670. 10.1177/19476035211046041 PMC8808919 34929763

[14] Eggeling L Breer S Drenck TC Frosch KH Akoto R. Double-layered quadriceps tendon autografts provide lower failure rates and improved clinical results compared with hamstring tendon grafts in revision ACL reconstruction. Orthop J Sports Med. 2021;9(12):23259671211046929. 10.1177/23259671211046929 PMC8652188 34901287

[15] Eggli S Kohlhof H Zumstein M , et al. Dynamic intraligamentary stabilization: novel technique for preserving the ruptured ACL. Knee Surg Sports Traumatol Arthrosc. 2015;23(4):1215-1221. 24651979 10.1007/s00167-014-2949-x PMC4371814

[16] Ferretti A Monaco E Vadalà A. Rotatory instability of the knee after ACL tear and reconstruction. J Orthop Traumatol. 2014;15(2):75-79. 23917728 10.1007/s10195-013-0254-y PMC4033809

[17] Filbay SR Culvenor AG Ackerman IN Russell TG Crossley KM. Quality of life in anterior cruciate ligament-deficient individuals: a systematic review and meta-analysis. Br J Sports Med. 2015;49(16):1033-1041. 26224582 10.1136/bjsports-2015-094864

[18] Georgoulis AD Ristanis S Moraiti CO , et al. ACL injury and reconstruction: clinical related in vivo biomechanics. Orthop Traumatol Surg Res. 2010;96(suppl 8):S119-S128. 10.1016/j.otsr.2010.09.004 21036116

[19] Glasbrenner J Raschke MJ Kittl C , et al. Comparable instrumented knee joint laxity and patient-reported outcomes after ACL repair with dynamic intraligamentary stabilization or ACL reconstruction: 5-year results of a randomized controlled trial. Am J Sports Med. 2022;50(12):3256-3264. 36005281 10.1177/03635465221117777 PMC9527444

[20] Hogan DW Burch MB Rund JM , et al. No difference in complication rates or patient-reported outcomes between bone-patella tendon-bone and quadriceps tendon autograft for anterior cruciate ligament reconstruction. Arthrosc Sports Med Rehabil. 2022;4(2):e417-e424. 10.1016/j.asmr.2021.10.019 PMC9042747 35494262

[21] Hoogeslag RAG Brouwer RW de Vries AJ Boer BC Huis In ‘t Veld R . Efficacy of nonaugmented, static augmented, and dynamic augmented suture repair of the ruptured anterior cruciate ligament: a systematic review of the literature. Am J Sports Med. 2020;48(14):3626-3637. 32101692 10.1177/0363546520904690

[22] Hoogeslag RAG Brouwer RW Huis In ‘t Veld R Stephen JM Amis AA . Dynamic augmentation restores anterior tibial translation in ACL suture repair: a biomechanical comparison of non-, static and dynamic augmentation techniques. Knee Surg Sports Traumatol Arthrosc. 2018;26(10):2986-2996. 29396585 10.1007/s00167-018-4848-z

[23] Hoogeslag RAG Huis In ‘t Veld R Brouwer RW de Graaff F Verdonschot N . Acute anterior cruciate ligament rupture: repair or reconstruction? Five-year results of a randomized controlled clinical trial. Am J Sports Med. 2022;50(7):1779-1787. 35486517 10.1177/03635465221090527

[24] Hootman JM Helmick CG Barbour KE Theis KA Boring MA. Updated projected prevalence of self-reported doctor-diagnosed arthritis and arthritis-attributable activity limitation among US adults, 2015-2040. Arthritis Rheumatol. 2016;68(7):1582-1587. 27015600 10.1002/art.39692 PMC6059375

[25] Johnston PT Feller JA McClelland JA Webster KE. Strength deficits and flexion range of motion following primary anterior cruciate ligament reconstruction differ between quadriceps and hamstring autografts. J ISAKOS. 2021;6(2):88-93. 33832982 10.1136/jisakos-2020-000481

[26] Kaeding CC Léger-St-Jean B Magnussen RA. Epidemiology and diagnosis of anterior cruciate ligament injuries. Clin Sports Med. 2017;36(1):1-8. 27871652 10.1016/j.csm.2016.08.001

[27] Karamchedu NP Murray MM Sieker JT , et al. Bridge-enhanced anterior cruciate ligament repair leads to greater limb asymmetry and less cartilage damage than untreated ACL transection or ACL reconstruction in the porcine model. Am J Sports Med. 2021;49(3):667-674. 33534613 10.1177/0363546521989265 PMC8099149

[28] Karpinski K Häner M Bierke S Diermeier T Petersen W. Comparing knee laxity after anatomic anterior cruciate ligament reconstruction using quadriceps tendon versus semitendinosus tendon graft. Orthop J Sports Med. 2021;9(7):23259671211014849. 10.1177/23259671211014849 PMC8312171 34368380

[29] Katz JN Arant KR Loeser RF. Diagnosis and treatment of hip and knee osteoarthritis: a review. JAMA. 2021;325(6):568-578. 33560326 10.1001/jama.2020.22171 PMC8225295

[30] Kwak YH Lee S Lee MC Han HS. Anterior cruciate ligament reconstruction with quadriceps tendon-patellar bone allograft: matched case control study. BMC Musculoskelet Disord. 2018;19(1):45. 29426312 10.1186/s12891-018-1959-0 PMC5807733

[31] Lai YS Chen WC Huang CH , et al. The effect of graft strength on knee laxity and graft in-situ forces after posterior cruciate ligament reconstruction. PLoS One. 2015;10(5):e0127293. 10.1371/journal.pone.0127293 PMC4441446 26001045

[32] Lansdown DA Riff AJ Meadows M Yanke AB Bach BR Jr. What factors influence the biomechanical properties of allograft tissue for ACL reconstruction? A systematic review. Clin Orthop Relat Res. 2017;475(10):2412-2426. 28353048 10.1007/s11999-017-5330-9 PMC5599386

[33] Lee DW Lee J Jang S , et al. Long-term outcomes of anterior cruciate ligament reconstruction using quadriceps tendon-patellar bone autograft. Orthop J Sports Med. 2021;9(6):23259671211017474. 10.1177/23259671211017474 PMC8193668 34179211

[34] Leister I Kulnik ST Kindermann H , et al. Functional performance testing and return to sport criteria in patients after anterior cruciate ligament injury 12-18 months after index surgery: a cross-sectional observational study. Phys Ther Sport. 2019;37:1-9. 30763887 10.1016/j.ptsp.2019.01.010

[35] Lind M Nielsen TG Soerensen OG Mygind-Klavsen B Faunø P. Quadriceps tendon grafts does not cause patients to have inferior subjective outcome after anterior cruciate ligament (ACL) reconstruction than do hamstring grafts: a 2-year prospective randomised controlled trial. Br J Sports Med. 2020;54(3):183-187. 31704697 10.1136/bjsports-2019-101000

[36] Luc B Gribble PA Pietrosimone BG. Osteoarthritis prevalence following anterior cruciate ligament reconstruction: a systematic review and numbers-needed-to-treat analysis. J Athl Train. 2014;49(6):806-819. 25232663 10.4085/1062-6050-49.3.35 PMC4264654

[37] Martin-Alguacil JL Arroyo-Morales M Martin-Gómez JL , et al. Comparison of knee sonography and pressure pain threshold after anterior cruciate ligament reconstruction with quadriceps tendon versus hamstring tendon autografts in soccer players. Acta Orthop Traumatol Turc. 2019;53(4):260-265. 31201076 10.1016/j.aott.2019.04.012 PMC6738273

[38] Martin-Alguacil JL Arroyo-Morales M Martín-Gomez JL , et al. Strength recovery after anterior cruciate ligament reconstruction with quadriceps tendon versus hamstring tendon autografts in soccer players: A randomized controlled trial. Knee. 2018;25(4):704-714. 29776815 10.1016/j.knee.2018.03.011

[39] Matsumoto A Yoshiya S Muratsu H , et al. A comparison of bone-patellar tendon-bone and bone-hamstring tendon-bone autografts for anterior cruciate ligament reconstruction. Am J Sports Med. 2006;34(2):213-219. 16282583 10.1177/0363546505279919

[40] Meyer CAG Gette P Mouton C Seil R Theisen D . Side-to-side asymmetries in landing mechanics from a drop vertical jump test are not related to asymmetries in knee joint laxity following anterior cruciate ligament reconstruction. Knee Surg Sports Traumatol Arthrosc. 2018;26(2):381-390. 28712025 10.1007/s00167-017-4651-2 PMC5794826

[41] Murray MM Fleming BC Badger GJ , et al. Bridge-enhanced anterior cruciate ligament repair is not inferior to autograft anterior cruciate ligament reconstruction at 2 years: results of a prospective randomized clinical trial. Am J Sports Med. 2020;48(6):1305-1315. 32298131 10.1177/0363546520913532 PMC7227128

[42] Murray MM Kalish LA Fleming BC , et al. Bridge-enhanced anterior cruciate ligament repair: two-year results of a first-in-human study. Orthop J Sports Med. 2019;7(3):2325967118824356. 10.1177/2325967118824356 PMC6431773 30923725

[43] Park HM Kim HS Lee YJ. Knee osteoarthritis and its association with mental health and health-related quality of life: a nationwide cross-sectional study. Geriatr Gerontol Int. 2020;20(4):379-383. 32037727 10.1111/ggi.13879

[44] Paterson KL Kasza J Hunter DJ , et al. Longitudinal association between foot and ankle symptoms and worsening of symptomatic radiographic knee osteoarthritis: data from the osteoarthritis initiative. Osteoarthritis Cartilage. 2017;25(9):1407-1413. 28506843 10.1016/j.joca.2017.05.002 PMC5565691

[45] Poehling-Monaghan KL Salem H Ross KE , et al. Long-term outcomes in anterior cruciate ligament reconstruction: a systematic review of patellar tendon versus hamstring autografts. Orthop J Sports Med. 2017;5(6):2325967117709735. 10.1177/2325967117709735 PMC5476329 28660230

[46] Sajovic M Stropnik D Skaza K. Long-term comparison of semitendinosus and gracilis tendon versus patellar tendon autografts for anterior cruciate ligament reconstruction: a 17-year follow-up of a randomized controlled trial. Am J Sports Med. 2018;46(8):1800-1808. 29741911 10.1177/0363546518768768

[47] Sajovic M Vengust V Komadina R Tavcar R Skaza K. A prospective, randomized comparison of semitendinosus and gracilis tendon versus patellar tendon autografts for anterior cruciate ligament reconstruction: five-year follow-up. Am J Sports Med. 2006;34(12):1933-1940. 16923826 10.1177/0363546506290726

[48] Schagemann J Koebrich T Wendlandt R , et al. Comparison of hamstring and quadriceps tendon autografts in anterior cruciate ligament reconstruction with gait analysis and surface electromyography. J Orthop Traumatol. 2021;22(1):20. 34021423 10.1186/s10195-021-00581-z PMC8140171

[49] Shani RH Umpierez E Nasert M Hiza EA Xerogeanes J. Biomechanical comparison of quadriceps and patellar tendon grafts in anterior cruciate ligament reconstruction. Arthroscopy. 2016;32(1):71-75. 26382635 10.1016/j.arthro.2015.06.051

[50] Sharifi M Shirazi-Adl A. Knee flexion angle and muscle activations control the stability of an anterior cruciate ligament deficient joint in gait. J Biomech. 2021;117:110258. 33493713 10.1016/j.jbiomech.2021.110258

[51] Smith AH Capin JJ Zarzycki R Snyder-Mackler L. Athletes with bone-patellar tendon-bone autograft for anterior cruciate ligament reconstruction were slower to meet rehabilitation milestones and return-to-sport criteria than athletes with hamstring tendon autograft or soft tissue allograft: secondary analysis from the ACL-SPORTS trial. J Orthop Sports Phys Ther. 2020;50(5):259-266. 31775553 10.2519/jospt.2020.9111 PMC7196003

[52] Strauss MJ Miles JW Kennedy ML , et al. Full thickness quadriceps tendon grafts with bone had similar material properties to bone-patellar tendon-bone and a four-strand semitendinosus grafts: a biomechanical study. Knee Surg Sports Traumatol Arthrosc. 2022;30(5):1786-1794. 34591124 10.1007/s00167-021-06738-x

[53] Sun K Tian SQ Zhang JH , et al. Anterior cruciate ligament reconstruction with bone-patellar tendon-bone autograft versus allograft. Arthroscopy. 2009;25(7):750-759. 19560639 10.1016/j.arthro.2008.12.023

[54] Tengman E Brax Olofsson L Nilsson KG , et al. Anterior cruciate ligament injury after more than 20 years: I. Physical activity level and knee function. Scand J Med Sci Sports. 2014;24(6):e491-e500. 10.1111/sms.12212 24673102

[55] Tian S Wang B Liu L , et al. Irradiated hamstring tendon allograft versus autograft for anatomic double-bundle anterior cruciate ligament reconstruction: midterm clinical outcomes. Am J Sports Med. 2016;44(10):2579-2588. 27466222 10.1177/0363546516655333

[56] Tian S Wang Y Wang B , et al. Anatomic double-bundle anterior cruciate ligament reconstruction with a hamstring tendon autograft and fresh-frozen allograft: a prospective, randomized, and controlled study. Arthroscopy. 2016;32(12):2521-2531. 27289276 10.1016/j.arthro.2016.04.013

[57] Webster KE Feller JA Hartnett N Leigh WB Richmond AK. Comparison of patellar tendon and hamstring tendon anterior cruciate ligament reconstruction: a 15-year follow-up of a randomized controlled trial. Am J Sports Med. 2016;44(1):83-90. 26578718 10.1177/0363546515611886

[58] Widner M Dunleavy M Lynch S. Outcomes following ACL reconstruction based on graft type: are all grafts equivalent? Curr Rev Musculoskelet Med. 2019;12(4):460-465. 31734844 10.1007/s12178-019-09588-w PMC6942094

[59] Wright RW. Osteoarthritis classification scales: interobserver reliability and arthroscopic correlation. J Bone Joint Surg Am. 2014;96(14):1145-1151. 25031368 10.2106/JBJS.M.00929 PMC4083772

[60] Xie X Xiao Z Li Q , et al. Increased incidence of osteoarthritis of knee joint after ACL reconstruction with bone-patellar tendon-bone autografts than hamstring autografts: a meta-analysis of 1,443 patients at a minimum of 5 years. Eur J Orthop Surg Traumatol. 2015;25(1):149-159. 24748500 10.1007/s00590-014-1459-3

[61] Yoo SH Song EK Shin YR Kim SK Seon JK. Comparison of clinical outcomes and second-look arthroscopic findings after ACL reconstruction using a hamstring autograft or a tibialis allograft. Knee Surg Sports Traumatol Arthrosc. 2017;25(4):1290-1297. 26718638 10.1007/s00167-015-3955-3

[62] Zhou Y Fuimaono-Asafo A Frampton C van Niekerk M Hirner M. Quadriceps tendon autograft is comparable to hamstring tendon and bone-patella-tendon-bone up to 2 years after isolated primary anterior cruciate ligament reconstruction. Knee Surg Sports Traumatol Arthrosc. 2023;31(8):3268-3276. 36894784 10.1007/s00167-023-07370-7

